# Validation of criterion-based patient assignment and treatment effectiveness of a multidisciplinary modularized managed care program for headache

**DOI:** 10.1007/s10194-012-0453-6

**Published:** 2012-05-13

**Authors:** Thomas-Martin Wallasch, Christiane Hermann

**Affiliations:** 1 Headache Center Berlin at the Sankt Gertrauden Krankenhaus Berlin, Berlin, Germany; 2 MEDAS Ostschweiz, Interdisciplinary Medicine, Kornhausstr. 3, 9000 St. Gallen, Switzerland; 3 Department of Clinical Psychology, Justus-Liebig University Giessen, Giessen, Germany

**Keywords:** Integrated care, Multidisciplinary modularized treatment program, Outcome study, Headache-related disability, Headache-related quality of life, Chronic headache

## Abstract

This prospective observational study evaluates the validity of an algorithm for assigning patients to a multidisciplinary modularized managed care headache treatment program. *N* = 545 chronic headache sufferers [migraine (53.8 %), migraine + tension type (30.1 %), tension type (8.3 %) or medication overuse headache (6.2 %), other primary headaches (1.5 %)] were assigned to one of four treatment modules differing with regard to the number and types of interventions entailed (e.g., medication, psychological intervention, physical therapy, etc.). A rather simple assignment algorithm based on headache frequency, medication use and psychiatric comorbidity was used. Patients in the different modules were compared with regard to the experienced burden of disease. 1-year follow-up outcome data are reported (*N* = 160). Headache frequency and analgesic consumption differed significantly among patients in the modules. Headache-related disability was highest in patients with high headache frequency with/without medication overuse or psychiatric comorbidity (modules 2/3) compared to patients with low headache frequency and medication (module 0). Physical functioning was lowest in patients with chronic headache regardless of additional problems (modules 1/2/3). Psychological functioning was lowest in patients with severe chronicity with/without additional problems (module 2/3) compared to headache suffers with no/moderate chronicity (module 0/1). Anxiety or depression was highest in patients with severe chronicity. In 1-year follow-up, headache frequency (minus 45.3 %), consumption of attack-aborting drugs (minus 71.4 %) and headache-related disability decreased (minus 35.9 %). Our results demonstrate the clinical effectiveness and the criterion validity of the treatment assignment algorithm based on headache frequency, medication use and psychiatric comorbidity.

## Introduction

Headache (HA) is one of the most common types of chronic pain. In adults, about 4 % experience HA episodes on a near daily or daily basis [1]. Chronic headache imposes an immense burden on the individual and society because of significant morbidity and indirect and direct costs. The annual value of lost productivity in the United States is estimated to reach $13 billion for migraine and $19 billion for all headache disorders [2, 3]. Headache-related disability (HRD) is reported by more than 85 % of migraineurs. Headaches reduce the quality of life, decrease social and job functioning, and increase utilization of health care systems [4, 5]. Accordingly, the World Health Organization (WHO) has ranked migraine among the top 20 most disabling medical diseases worldwide [6].

Despite advances in the acute and preventive treatment of primary headaches, many headache patients remain misdiagnosed and undertreated [7–10]. Headache sufferers are often dissatisfied with the health care they receive. On average, patients with chronic headache utilize more health care resources than patients with other chronic diseases, for example consuming twice as much medication [11, 12].

Cross-sectional and longitudinal epidemiological studies as well as clinical studies, family and twin studies have repeatedly reported a high comorbidity between headache and mood and anxiety disorders. Psychiatric disorders may interfere with headache treatment. HA patients with a comorbid mood or anxiety disorders report lower health-related quality of life (HRQoL) [13, 14], have a poorer prognosis and respond less well to treatment. The prevalence and impact of psychiatric disorders in headache patients underline the necessity for screening patients as part of routine clinical care and not only of refractory patients in tertiary headache centers [15].

In clinical practice, there is a lack of multidisciplinary headache treatment programs that entail a comprehensive assessment including a headache diagnosis according to ICDH-II criteria [16], screening for psychiatric comorbidity, craniomandibular dysfunctioning and musculoskeletal disorders, and provide treatment according to clinical guidelines [17, 18]. Unfortunately, little is known about such integrated headache programs and the patients participating in such programs [19, 20].

In 2005, Diener and co-workers launched the West German Headache Center, a managed and modularized healthcare system for chronic headache patients [21, 22] that was rated by the Harvard business school as one of the best medical programs worldwide [23]. Later, centers in Berlin, Munich and Jena followed. Primarily, this paper focuses on the validity of patient assignment to the treatment modules of our integrated headache care program. Specifically, patients in the different modules were compared with regard to pain-related disability, health-related quality of life and psychological distress. For a subset of patients, we also report 1-year follow-up data of patients with frequent or difficult to treat headaches. Detailed data on the program’s cost-effectiveness have been reported elsewhere [24].

## Methods

### The Headache Center Berlin (HCB)

The Headache Center Berlin started in 07/2006 as a tertiary outpatient headache clinic. Treatment is multidisciplinary with daily treatment sessions. Additional inpatient treatment facilities (5 beds) are available for patients with medication overuse and severe psychiatric comorbidity. The HCB cooperates with a network of primary care physicians and headache specialists (secondary care). All network partners are connected with the HCB by specifically designed online documentation software [25]. At the HCB, European Headache Federation guidelines for the organization of headache clinics are implemented [26].

### Study design

This prospective study was done to evaluate the new treatment approach used in the HCB. Adult patients (>18 years) with frequent and\or difficult to treat headache diagnoses of migraine (*M*), tension-type headache (TTH), and medication overuse headache (MOH) were consecutively enrolled within a time period of 18 months (11/2006–04/2008). Headache diagnoses were made according to ICHD-II criteria [16] by a board-certified neurologist (TMW). The baseline data of the total cohort were used for determining the validity of treatment assignment. A representative subgroup of these patients completed treatment during this time period. For these, we report the 1-year follow-up data. The remaining patients followed the treatment program. A reduction of headache frequency (days with headache/month) of >50 % was defined as treatment success and served as primary outcome parameter. Secondary outcomes were changes in headache-related disability, anxiety and depression and analgesic use. All patients signed a written informed consent form. The project was approved by the local Ethics Committee.

### Assessment

Patients were referred by physicians or specialists when headache treatment failed. In addition, health insurance companies identified eligible patients on the basis of inpatient data, sick leave or records of prescribed medication. Finally, self-referrals were encouraged by media coverage. Patients kept a headache diary for at least 4 weeks prior to the first study visit. All patients underwent an initial comprehensive assessment by a neurologist, a psychologist and a physical therapist, and completed several questionnaires assessing mood, headache-related disability and health-related quality of life (see below for details). A detailed headache history was taken, patients underwent a physical exam, and a HA diagnosis was made according to ICHD-II criteria [16]. If necessary, additional diagnostic tests (imaging, blood test, etc.) were run. The psychologist obtained information about the patient’s level of stress, emotional well-being, job satisfaction, life events, and data on possible psychological HA triggers were collected. Mental disorders were assessed based on ICD-10 [27], which is the standard classification system used in the German health system. A physical therapist examined posture and muscle function. Subsequent to the assessment, both team and patient met in a pain conference and made a joint decision about further treatment.

### Treatment

As stated earlier, the managed care system entails a modularized treatment protocol. Patients were assigned to one of four treatment modules taking into consideration headache frequency, medication overuse and psychiatric comorbidity. Typically, treatment duration is 1 year.

### Module 0: no or little chronicity

Patients assigned to module 0 have a maximum of 5 HA days/month. Patients are referred back to primary care physicians with recommendation for improved headache management (e.g. change of medication).

### Module 1: moderate chronicity

Patients with a HA frequency between 6 and <10 HA days/month and <10 days with intake of analgesics/triptans are assigned to this module. Treatment includes education and patient self-management for preventing headache episodes. Medication is optimized if necessary.

### Module 2: severe chronicity

Patients with more than 10 HA days/month and more than 10 days with intake of analgesics/triptans are assigned to module 2. Patients receive module 1 treatment with a maximum of 12 additional outpatient sessions on 5 consecutive days from 9.00 a.m. to 4.00 p.m. The program entails group sessions and individual appointments with the neurologist, psychologist and the physical therapist. The neurologist provides headache education (90 min per day). The psychologist provides cognitive-behavioral pain management. The physical training comprises endurance sport, physical therapy and Nordic walking (60 min per day).

### Module 3: severe chronicity with additional problems

Patients with more than 15 HA days/month and more than 15 days with intake of analgesics/triptans and severe psychiatric comorbidity are assigned to this module. Aside from participating in the outpatient program of module 2, they are hospitalized for 5 up to a maximum of 7 days and undergone drug withdrawal. Treatment entails initiating adequate acute and prophylactic drug management.

## Study patients

During a time period of 18 months (11/2006–04/2008), a cohort of 545 headache sufferers was consecutively referred to the HCB, which started in 07/2006 with a new modularized headache managed care program. Baseline data of these patients (*N* = 545) were available for validation of criterion-based patient assignment to the present modularized headache treatment program. With the exception of patients suffering from medication overuse headache, patients with secondary headaches were excluded. Of the total cohort, *N* = 83 patients were assigned to module 0, *N* = 158 patients to module 1, *N* = 249 to module 2 and *N* = 55 patients to module 3.

During the 18-month study period *N* = 160 patients completed the treatment, while the remaining patients followed treatment program. For these, 12-month follow-up outcome data are reported. Of these, *N* = 47 (29.4 %) had been assigned to module 1, *N* = 97 (60.6 %) to module 2 and *N* = 16 (10 %) to module 3.

## Questionnaires

All questionnaires were administered on a pocket PC using AC-STB software from Akkaya company, Köln, Germany [24, 25].

### Headache-related disability

The Migraine Disability Assessment (MIDAS) Questionnaire [28, 29] was developed to assess headache-related disability. It contains five questions assessing the number of missed days (full or with substantial loss of productivity) at work, household chores, and leisure time. Reliability and validity of the MIDAS have extensively been demonstrated [28–30]. The total MIDAS score can be used to grade migraine-related disability [grade 1 (minimal disability): 1–5 points, grade 2 (mild): 6–10 points, grade 3 (moderate): 11–20 points, grade 4 (severe): >20 points] [28]. Headache frequency (past 3 months) and intensity are assessed by two additional MIDAS items.

### Chronic Pain Grade Questionnaire (CPGQ)

The CPGQ [31] consists of 6 items assessing pain intensity or disability on 11-point numerical rating scales ranging from “0” to “10”. In addition, the number of days with disability during the past 3 months is obtained. Using an empirically derived and validated grading system, severity is divided into five categories: grade 0 (pain free), grade 1 (low disability, low-pain intensity), grade 2 (low disability, high-pain intensity); grade 3 (high disability, moderately limiting pain); and grade 4 (high disability, severely limiting pain). Reliability and validity of the CPGQ have been shown for the German version [32].

### Health-related quality of life (HRQoL)

Health-related quality of life was assessed using the German version of the 12 item-Short Form Health Survey (SF12 [33], German version: [34]). The SF12 contains 2 subscales of functioning (“physical”/“psychological”). The SF12 is reliable and valid, norms for the general population are available [34].

### Anxiety and depression

The Hospital Anxiety and Depression Scale (HADS, (German: [35]) contains seven items each for assessing depression and anxiety. The HADS is recommended for patients with somatic problems [14]. In its original version, a score between 0 and 7 is considered to be in the normal range, a score between 8 and 10 as being slightly elevated, and a score of 11 and higher as indicating the probable presence of a mood or anxiety disorder.

## Data analysis

Depending on the type of outcome variable, differences between modules were computed either with one-way analysis of variances (ANOVA) followed by Tukey’s HSD post hoc tests for all pairwise contrasts or by chi-square tests. Treatment data were computed either with Student *t* test or Mann–Whitney *U* test when variables were not normally distributed. All analyses were performed using Predictive Analytics SoftWare (PASW) by SPSS. A* p* value of 0.05 was considered as significant.

## Results

### Study sample

Table 1 shows sociodemographics, diagnoses and headache characteristics at baseline of the total cohort. There were no significant differences in headache diagnoses, headache characteristics and sociodemographics of the subgroup of treated patients in comparison with the total cohort.

**Table 1:** Sociodemographic data and clinical characteristics of all headache patients

	Total (*N* = 545)	Module 0 (*N* = 83)	Module 1 (*N* = 158)	Module 2 (*N* = 249)	Module 3 (*N* = 55)
Age (year; *M*, SD)	43.08 (12.94)	42.70 (13.15)	43.94 (12.61)	42.50 (12.89)	43.90 (13.94)
*N*, females (%)	489 (89.7)	77 (92.8)	145 (91.8)	222 (89.2)	45 (81.8)
HA diagnosis (%)					
Migraine^A^	293 (53.8)	55 (66.3)	90 (57.0)	118 (47.4)	30 (54.5)
Tension-type HA (%)^A^	43 (8.3)	3 (3.6)	6 (3.8)	32 (12.9)	4 (7.3)
Migraine + TTH (%)^A^	164 (30.1)	21 (25.3)	55 (34.8)	73 (29.3)	15 (27.3)
Migraine + other HA disorders (%)^B^	13 (2.4)	1 (1.2)	1 (0.6)	10 (4.0)	1 (1.8)
TTH + other HA disorders (%)^B^	3 (0.6)	0 (0)	0 (0)	2 (0.8)	1 (1.8)
Migraine + TTH + other HA disorders (%)^B^	1 (0.2)	1 (1.2)	0 (0)	0 (0)	0 (0)
Other HA disorders (%)^B^	16 (2.9)	1 (1.2)	5 (3.2)	9 (3.6)	1 (1.8)
MOH only (%)^C^	8 (1.5)	0 (0)	0 (0)	5 (2.0)	3 (5.5)
MOH (w/o other HA disorders; %)^D^	23 (4.2)	0 (0)	3 (1.9)	13 (5.2)	7 (12.7)
HA duration (year; M, SD)	20.69 (12.65)	19.85 (13.01)	22.09 (12.28)	19.59 (12.92)	22.86 (11.52)
MIDAS (M, SD)					
Headache frequency (last 3 months)	28.43 (23.11)	8.45^a^ (3.76)	22.39^b^ (9.62)	35.59^c^ (25.66)	43.55^d^ (29.64)
Headache intensity (last 3 months)	6.37 (2.00)	6.40^a^ (2.12)	6.42^a^ (1.82)	6.32^a^ (2.07)	6.42^a^ (2.04)
Analgesic use (*N* days/month, M, SD)	8.84 (10.24)	4.48^a^ (4.91)	6.47^a^ (6.96)	9.13^b^ (8.93)	20.85^c^ (17.9)
Medication doses (*N* per months; M, SD)	18.77 (28.58)	8.99^a^ (4.91)	12.96^a^ (10.8)	17.53^a^ (17.1)	55.8^b^ (69.34)

Differences between modules were computed either with one-way analysis of variances (ANOVA [49]) or with χ^2^ tests, depending on the type of variable. Means with different lowercase superscripts differ significantly (Tukey’s HSD)

*HA* headache, *MOH* medication overuse headache, *TTH* tension-type HA

^+^Headache diagnosis was missing for two patients, one each in modules 0 and 1. Hence, percent values do not add up to 100

^A^With or without MOH

^B^Other HAs refer to all HA diagnoses except MOH

^C^Underlying primary headache not definable

^D^This refers to the total number of patients with an MOH diagnosis in the total sample or the modules

### Comparison of modules

There was no significant difference between patients in the four modules with regard to age (*p* > 0.5) and sex proportion (*p* > 0.15). The vast majority of patients suffered from migraine alone or in combination with tension-type or other headaches. For the past 3 months, patients reported on average a headache frequency of 28.43 ± 23.11 days which differed significantly across groups (F(3,541) = 51.16, *p* < 0.001). As illustrated in Table 1 and confirmed by significant pairwise post hoc contrasts, headache frequency increased significantly from modules 0 to 1, 1 to 2, and 2 to 3. Headache intensity was on average 6.37 ± 2.00 with no significant difference between modules (*p* > 0.9). Patients relied on analgesics on an average of 8.84 ± 10.24 days per month. Analgesic uses varied significantly across modules (F(3,539) = 40.35, *p* < 0.001) with patients in modules 0 and 1 using significantly less analgesics than patients in modules 2 and 3, and patients in module 3 having a significantly more frequent use than patients in module 2 (see Table 2). Patients reported an average consumption of medication doses of 6.34 ± 10.15 per month which varied significantly across the modules (F(3,539) = 44.14, *p* < 0.001) with patients in module 3 using the greatest amount of medication compared to all other patients. Patients in modules 0, 1 and 2 did not significantly differ with regard to the number of medication doses.

**Table 2:** Pain-related disability (MIDAS, von Korff index)

	Total	Module 0	Module 1	Module 2	Module 3
MIDAS					
Total score (*M*, SD)	43.43 (42.44)	18.49^a^ (17.20)	33.77^b^ (29.12)	53.9^c^ (48.52)	61.42^c^ (48.55)
Grade 1 (*N*, %)	63 (11.6)	19 (22.9)	16 (10.1)	26 (10.4)	2 (3.6)
Grade 2 (*N*, %)	58 (10.6)	15 (18.1)	21 (13.3)	16 (6.4)	6 (10.9)
Grade 3 (*N*, %)	79 (14.5)	22 (26.5)	21 (13.3)	32 (12.9)	4 (7.3)
Grade 4 (*N*, %)	345 (63.3)	27 (32.5)	100 (63.3)	175 (70.3)	43 (78.2)
CPGQ (von Korff)					
Grade 1 (*N*, %)	138 (25.3)	39 (47.0)	43 (27.2)	50 (20.1)	6 (10.9)
Grade 2 (*N*, %)	122 (22.4)	21 (25.3)	36 (22.8)	56 (22.5)	9 (16.4)
Grade 3 (*N*, %)	212 (38.9)	22 (26.5)	63 (39.9)	97 (39.0)	30 (54.5)
Grade 4 (*N*, %)	73 (13.4)	1 (1.2)	16 (10.1)	46 (18.5)	10 (18.2)

Differences between modules were computed either with one-way analysis of variances (ANOVA [49]) or χ^2^ tests, depending on the type of variable. Means with different superscripts differ significantly (Tukey’s HSD)

### Headache-related disability

The MIDAS total score differed significantly between modules (F(3,541) = 23.14, *p* < 0.001). Post hoc Tukey contrasts revealed that patients in module 0 had a significantly lower MIDAS total score compared to all other patients. Patients in module 1 had significantly lower MIDAS total scores than patients in modules 2 and 3, with the latter two not differing significantly (see Table 2). Consistently, the percentage of patients having a MIDAS grade III or IV increased significantly from module 0 to module 3 (χ^2^(9) = 49.4, *p* < 0.001; see Table 2).

### Chronic Pain Grading Questionnaire (von Korff index)

Similar to the MIDAS grades, the von Korff grades of severity differed significantly between modules (χ^2^(9) = 47.03, *p* < 0.001; see Table 2). The proportion of patients with a severity grade of III or IV increased from modules 0 (27 %) to 3 (72 %) with modules 1 (50 %) and 2 (57 %) lying in between.

### Anxiety and depression

One-way ANOVAs revealed significant differences with regard to HADS anxiety (F(3,541) = 23.84, *p* < 0.001) and depression scores (F(3,541) = 22.94, *p* < 0.001). Post hoc contrasts (Tukey HSD) showed that anxiety and depression levels of patients in modules 0 and 1 were not significantly different. However, both groups reported significantly less anxiety and depressive symptoms than patients in modules 2 and 3, with the latter not differing significantly. Correspondingly, the percentage of patients with a HADS anxiety (χ^2^(3) = 40.06, *p* < 0.001) or depression score (χ^2^(3) = 23.34, *p* < 0.001) above the cutoff of 11 differed significantly between modules, with the patients in modules 2 and 3 being most likely to have an anxiety or mood disorder (see Table 3).

**Table 3:** Anxiety, depression and health-related quality of life (SF12) (*M*; SD) in the patients assigned to the four modules

	Total	Module 0	Module 1	Module 2	Module 3
HADS—Anxiety					
Total score (*M*, SD)	7.13 (3.95)	5.23^a^ (2.9)	5.85^a^ (3.35)	8.35^b^ (3.99)	8.18^b^ (4.53)
*T* score (*M*, SD)	54.85 (11.4)	49.46 (9.68)	51.23 (10.58)	58.34 (10.94)	57.57 (12.00)
Above cutoff (*N*, %)	114 (20.9)	4^a^ (4.8)	17^b^ (10.8)	76^c^ (30.5)	18^d^ (32.7)
HADS—depression					
Total score (*M*, SD)	5.57 (3.93)	3.7^a^ (2.52)	4.31^a^ (3.52)	6.69^b^ (4.06)	6.91^b^ (4.12)
T score (*M*, SD)	59.99 (11.48)	54.55 (8.89)	56.13 (11.2)	63.37 (10.99)	64.00 (11.46)
Above cutoff (*N*, %)	67 (12.3)	1^a^ (1.2)	17^b^ (10.8)	44^c^ (17.7)	11^d^ (20.0)
SF-12 (*M*, SD)					
Physical	40.02 (8.00)	43.62^a^ (6.50)	40.35^b^ (7.62)	39.08^b^ (8.24)	37.88^b^ (8.47)
Psychological	45.08 (10.62)	50.13^a^ (8.33)	40.08^a^ (9.40)	42.07^b^ (10.97)	42.47^b^ (10.38)

Differences between modules were computed either with one-way analysis of variances (ANOVA [49]) or χ^2^ tests, depending on the type of variable. Means with different superscripts differ significantly (Tukey’s HSD)

*HADS* Hospital Anxiety and Depression Scale, *SF-12* 12 item version of the Short Form of Medical Outcomes Questionnaire

### Health-related quality of life

With regard to physical HQoL, patients in module 0 reported a significantly higher level compared to the patients in all other modules, with no significant difference between modules 1, 2 and 3 (F(3,541) = 8.45, *p* < 0.001; see Table 3). With regard to psychological HQoL, the one-way ANOVA also revealed a significant group main effect (F(3,541) = 20.18, *p* < 0.001). Similar to the findings obtained for the HADS, the post hoc Tukey HSD contrasts showed that patients in modules 0 and 1 reported a similar level of psychological HQoL which was significantly lower than in modules 2 and 3 patients which, in turn, were not significantly different.

### One-year follow-up

Mean reduction in HA frequency was 6.8 days/month. Headache frequency decreased from 15.00 ± 8.19 to 8.22 ± 8.63 (−45.3 %) days/month (*t* = 5.056, *df* = 157, *p* < 0.001). 61.1 % of the patients (96/157) experienced a reduction of HA frequency (*N* HA days per month) of greater than 50 %. Headache-related disability (MIDAS) decreased significantly from 55.96 ± 61.28 to 35.87 ± 55.09 (*t* = 4.924, *df* = 129, *p* < 0.001). HADS anxiety was also significantly lower at the 1-year follow-up (pre: *M* = 7.60 ± 4.24; follow-up: *M* = 6.66 ± 4.24; *t* = 2.567,*df* = 91, *p* = 0.012). The level of depressive symptoms (HADS) did not change significantly (pre: *M* = 5.70 ± 3.77; follow-up: *M* = 5.10 ± 4.54). Medication (analgesics, triptans, ergotamine) was used by 98.7 % of the patients at baseline and by 89.9 % of patients at the 1-year follow-up with a highly significant reduction in the number of days with medication use per month (pre: *M* = 10.92 ± 7.31; follow-up: 3.99 ± 3.62; (*t* = 9.686, *df* = 157, *p* < 0.000). At baseline, 24 % of the patients reported an intake frequency of ≥15 days/month. 57 % of patients used acute medication on 6–14 days/month and 17.7 % on 1–5 days/month. At the end of treatment, only 1.3 % of the patients consumed acute medication on ≥15 days/month, 23.4 % relied on acute medication on 6–14 days/month. About two-thirds of the patients (65.2 %) used acute medication on 1 up to 5 days/month. The number of patients who abstained from acute medication increased from 1.3 to 10.1 %. The number of patients who abstained from acute medication increased significantly from 1.3 % at baseline to 10.1 % at the end of treatment [χ^2^ (61.348) = 43.4; *df* = 9; *p* < 0.001].

## Discussion

Providing efficient multidisciplinary treatment for difficult to treat patients is a challenge. Few data exist on how to assign patients to treatment and on effectiveness of such a multidisciplinary integrated headache care program for patients with frequent HAs, including MOH. A modularized program was chosen, because this allows to tailor the treatment to an individual’s needs, and because it is not the referring physician who assigns the patient to specific interventions but treatment assignment is based on a multidisciplinary assessment.

The primary goal of the present study was the validation of a criterion-based patient assignment to the modules of our integrated headache care program. Our results document that the algorithm used to assign the patients to treatment modules specifically designed to meet their need works. A main aim was to offer managed health care for out- and inpatients that is tailored individually using HA frequency, analgesic consumption, and psychiatric comorbidity as criteria for treatment indication. Most of the available interdisciplinary headache concepts only offer a standardized program which does not allow an individual adjustment based on patients’ needs [36, 37]. We developed a modularized headache treatment program with the aim of treating chronic headache suffers according their burden of disease. Patients are assigned to treatment modules based on headache frequency, medication overuse and psychiatric comorbidity. Most crucially, it needs to be shown that this algorithm allows a valid patient assignment with regard to headache-related disability and quality of life. As was to be expected when HA frequency is used as assignment criterion, HA frequency rose significantly from modules 0 to 3 with a significant increase between each module. By contrast, HA intensity was comparable in all patients. Similarly, analgesic consumption differed significantly in the four modules. Patients assigned to module 3 reported significantly more days of analgesic consumption and medication doses per month than those in the other modules. Patients in module 2 relied on medication on significantly fewer days per month than those in module 3, but on significantly more days than those in modules 0 and 1. The total number of medication doses per month did not differ significantly between modules 0, 1 and 2. These results show that the patients’ assignment based on HA frequency and medication use was well efficient.

Importantly, we aimed at validating patients’ assignment to the different treatment modules with regard to external criteria, such as headache-related disability and health-related quality of life. The average MIDAS total score was 43.3, ranging from 18.5 in module 0 to 61.4 in module 3. Overall, these MIDAS scores well match those previously reported for chronic headache sufferers [38, 39]. Disability in patients assigned to module 2 or 3 was comparable. However, these patients were significantly more disabled than patients in modules 0 and 1, with patients in module 0 being also significantly less disabled than patients in module 1. Correspondingly, the percentage of patients having a MIDAS grade III or IV increased significantly from module 0 to module 3. A rather similar pattern was observed with regard to the von Korff chronic pain grade questionnaire. Hence, both MIDAS and CPGQ scores were highest in patients assigned to module 3 and lowest in patients assigned to module 0. Module 2 patients’ disability was comparable to module 3 patients’ if the MIDAS score was considered and comparable to module 1 patients’ if the CPGQ score was used. This difference is most likely accounted for by the fact that MIDAS measures disability primarily based on the number of days of lost productivity (e.g., work), whereas the CPGQ relies on the perceived degree of disability. Given that patients are assigned to module 3 rather than 2 if there is psychiatric comorbidity and not due to differences in HA activity and use of medication, it is rather plausible that disability determined based on the number of lost days due to HA does not significantly differ between modules 2 and 3. Consistent with the findings for HA disability, health-related quality of life also varied between modules. With regard to the SF12 physical scale, patients with no or little chronicity (module 0) reported a significantly higher level of health-related quality of life compared to the patients in all other modules. By contrast, psychological functioning (SF12 mental health scale) was highest in patients assigned to modules 0 (no or less chronicity) and 1 (moderate chronicity) and lowest in patients in modules 2 (severe chronicity) and 3 (severe chronicity with additional problems). This pattern suggests that a HA frequency of more than 5 days/month is associated with a distinct reduction in physical functioning, whereas psychological quality of life is strongly affected when HA frequency exceeds 10 days per month. However, there is increasing awareness that mood and anxiety disorders are positively correlated with chronification of headache [40–42]. The observed decrease in psychological functioning is most likely accounted for by elevated anxiety and depression levels. Such an interpretation is supported by the observed pattern of the anxiety and depression HADS scores. Patients in modules 0 and 1 had similar anxiety and depression levels and a similar number of patients had HADS scores above the cutoff. These patients were significantly less anxious and depressed than the patients in modules 2 and 3, with no significant difference between them. Clearly, we had expected that patients assigned to module 3 would be significantly more anxious and depressed than all other patients, given that psychiatric comorbidity as assessed by a clinical psychologist is a primary criterion for a patient’s assignment to module 3. Our findings suggest that the HADS was suitable for detecting patients with clinically elevated levels of anxiety and depression. This has important clinical implications as it suggest that patients with frequent HAs should be routinely screened using the HADS. The observed relationship between high HA frequency, high use of analgesics, high pain-related disability and high emotional distress is consistent with previous observations that psychiatric comorbidity contributes to the chronicity and intractability of headache [41, 43].

A second aim of the present study was to provide preliminary data on the effectiveness of this modularized HA treatment program. In a subset of patients, the primary and secondary outcome variables improved significantly. Specifically, mean HA frequency decreased by about 6.8 days/month. On average, HA frequency was reduced by 45.3 %. This success rate is similar to previous studies which reported improvements of about 35 % (e.g. Lemstra et al. [44]: 33.6 % at 3-month follow-up; Gaul et al. [21]: 34.3 % at a follow-up time between 12 and 18 months after the end of treatment). The number of days with acute medication intake, known as predictor of MOH [45], was also significantly reduced. The percentage of patients who relied on non-pharmacological self-management strategies increased from about 1.3 % at baseline to 10.1 % after 1 year. At follow-up, only 1.3 % of the patient used medication on more than 15 days per month, at baseline 24 % of the patients showed this pattern of medication use. This reduction is impressive, because previous studies have reported relapse rates of drug withdrawal of up to 40 % within 1 year [46–48].

Taken together, our study shows that using a simple algorithm, it is feasible to assign HA patients to treatment modules tailored to patients’ needs. Such a treatment assignment to a modularized treatment program seems promising for an effective and cost-efficient treatment of chronic HA patients.
